# Identifying Key Drivers of Foodborne Diseases in Zhejiang, China: A Machine Learning Approach

**DOI:** 10.3390/foods14162857

**Published:** 2025-08-18

**Authors:** Cangyu Jin, Xiaojuan Qi, Jikai Wang, Lili Chen, Jiang Chen, Han Yin

**Affiliations:** 1 Department of Nutrition and Food Safety, Zhejiang Provincial Center for Disease Control and Prevention, Hangzhou 310051, China; 2 School of Management, Zhejiang University, Hangzhou 310058, China

**Keywords:** foodborne disease, big data, machine learning, Random Forest, XG Boost, public health

## Abstract

Foodborne diseases represent a significant public health challenge worldwide. This study systematically analyzed the temporal dynamics, key predictors, and seasonal patterns of pathogen-specific foodborne diseases using a dataset of 56,970 cases from Zhejiang Province, China, spanning 2014 to 2023. A comprehensive set of 91 candidate variables was constructed by integrating epidemiological, environmental, socioeconomic, and agricultural data. Lasso regression was employed to identify 41 important predictors. Based on these variables, supervised machine learning models (Random Forest and XGBoost) were trained and evaluated, achieving training set classification accuracies of 86% and 87%, respectively, demonstrating robust performance. Feature importance analysis revealed that patient age, food type, climate policy, and processing methods were the most influential determinants, highlighting the combined impact of host, exposure, and environmental factors on disease risk. The results demonstrated significant shifts in the pathogen spectrum over the past decade, including a steady decline in Vibrio parahaemolyticus, an increase in Salmonella after 2016, and persistent seasonal peaks in Norovirus and Vibrio parahaemolyticus during warmer months. Seasonal ARIMA modeling and time-series decomposition further confirmed the critical role of seasonal and trend components in bacterial incidence. Overall, this study demonstrates the value of integrating machine learning and time-series analysis for pathogen-specific surveillance, risk prediction, and targeted public health interventions.

## 1. Introduction

Foodborne diseases represent a growing global health concern, with millions of cases reported annually, impacting public health systems and economies across various regions [1,2,3]. In China, where rapid economic development has led to changes in food production, distribution, and consumption patterns, foodborne illnesses remain a significant issue [4,5]. In recent years, with the intensification of uncertainties such as climate change, region-specific risks of foodborne diseases have become increasingly prominent [6]. Located on the eastern coast of China, Zhejiang Province is characterized by a dense population, a vibrant food industry, and diverse dietary practices, making it a hotspot for foodborne illness outbreaks. Its unique socio-economic landscape presents a range of challenges for food safety governance. The rapid pace of urbanization, high volumes of tourism, and growing demand for diversified food products have collectively heightened the risk of foodborne disease transmission. Particularly during the summer months, the province’s hot and humid climate creates favorable conditions for the proliferation and spread of foodborne pathogens. These environmental factors, combined with inadequate food storage and improper handling practices, further exacerbate the risk of contamination. Such a complex interplay of environmental and behavioral drivers positions Zhejiang Province as a valuable case for investigating regionally specific patterns and determinants of foodborne disease risk [7,8].

Between 2014 and 2023, Zhejiang Province recorded 57,474 cases of foodborne diseases. These cases provide a valuable basis for analyzing the specific factors influencing foodborne disease outbreaks in the region. Existing research has already identified key drivers of foodborne illnesses globally, such as microbial contamination, poor sanitation, and unsafe food practices; however, localized factors specific to Zhejiang remain underexplored. Understanding the regional dynamics, such as the influence of food supply chain complexities, seasonal fluctuations, and regional food preferences, is critical for developing targeted interventions.

Zhejiang Province, located on the southeast coast of China, is home to over 65 million residents, with a well-developed urban infrastructure. The province boasts one of the highest GDP per capita levels in China, driven by dynamic sectors such as manufacturing, trade, agriculture, and a burgeoning food processing industry. Zhejiang’s dietary culture features a mix of traditional fresh foods, such as seafood, rice, and vegetables, and an increasing consumption of processed and ready-to-eat products, reflecting broader national trends in food system transformation. Annual average temperatures in Zhejiang range from 16 °C to 19 °C, with humid subtropical conditions and abundant rainfall (averaging over 1500 mm per year), creating favorable environments for foodborne pathogen growth, particularly in summer months. The province’s food supply chain is characterized by both local production and substantial imports from other regions, further increasing complexity and exposure risk.

This study aims to bridge that gap by analyzing the factors that contributed to foodborne diseases in Zhejiang over the ten-year period. The research will focus on identifying patterns and trends within the dataset, examining how environmental conditions, food production practices, and consumer behaviors intersect to influence food safety outcomes. Furthermore, this analysis will explore the effectiveness of existing food safety regulations and public health interventions, with the objective of proposing data-driven recommendations for mitigating future outbreaks. By identifying the most significant risk factors in Zhejiang, this research seeks to inform more localized food safety policies and contribute to the broader understanding of foodborne disease prevention in China and beyond.

Foodborne diseases are influenced by a variety of factors, including microbial contamination, environmental conditions, and human behavior. Global literature has extensively explored the impact of foodborne pathogens, with bacteria such as Salmonella, Escherichia coli, and Listeria identified as major causative agents. These pathogens are often associated with improper hygiene practices during food processing, cross-contamination, and inadequate food storage [6]. Environmental factors, particularly temperature and humidity, are also widely recognized as critical elements that promote pathogen growth, especially under conditions where food is poorly stored or handled [9,10].

Studies have shown that foodborne diseases often exhibit seasonal patterns. Specifically, higher rates of illness are observed during summer months, when elevated temperatures accelerate microbial reproduction, increasing the likelihood of disease outbreaks [4,11]. For example, in many regions of China, including Zhejiang Province, the incidence of foodborne diseases is significantly higher in the summer. Additionally, improper food handling practices in households and the food service industry have been found to significantly increase the risk of foodborne illnesses [12,13].

Socioeconomic factors also play a significant role in the spread of foodborne diseases. Rapid urbanization, the growing demand for ready-to-eat foods, and the increasing complexity of the food supply chain have heightened the risk of contamination [9,10,14]. In Zhejiang Province, rapid urban development, alongside the expansion of the food industry and changes in consumer habits, has created more opportunities for foodborne disease outbreaks. Furthermore, small- and medium-sized enterprises (SMEs) in the food sector, due to regulatory shortcomings and inadequate enforcement of food safety standards, are often cited as potential sources of foodborne disease risk [9,15,16].

In terms of food safety regulations, while China has implemented various safety standards for food production and distribution, challenges remain in the enforcement of these standards, particularly in smaller enterprises. These challenges contribute to the persistence of food safety hazards [17,18]. Additionally, a lack of consumer awareness about food safety further exacerbates the risk of foodborne disease outbreaks [19,20].

In the context of international research, numerous studies have confirmed that climate, food supply chain complexity, socioeconomic development, and population dietary structure are key risk factors shaping the epidemiology of foodborne diseases in diverse regions [11,12,13], localized factors specific to Zhejiang Province which remain underexplored. For example, the role of Zhejiang’s climate, dietary habits, and the complexity of the regional food supply chain in influencing foodborne disease outbreaks requires further research. Moreover, the effectiveness of public health interventions and government regulations at that level has not been sufficiently evaluated. This gap in the literature highlights the need for more localized studies to analyze these complex factors and inform targeted foodborne disease prevention policies in Zhejiang Province.

While the existing literature has made significant progress in understanding the common causes and contributing factors of foodborne diseases, a clear research gap exists in the localized analysis of these factors in Zhejiang Province. Most studies focus on global or national trends, often overlooking the specific conditions in Zhejiang, such as its climate, dietary habits, and the complexity of its regional food supply chain. Furthermore, there is limited research evaluating the effectiveness of existing food safety regulations and public health interventions at the local level. Therefore, this study aims to fill this gap by analyzing the factors influencing foodborne diseases in Zhejiang Province from 2014 to 2023, providing data-driven insights for the development of more effective and targeted prevention strategies.

## 2. Data

### 2.1. Data Source

We utilized foodborne disease surveillance data from Zhejiang Province, China, spanning the years 2014 to 2023. The dataset was obtained from the Zhejiang Provincial Center for Disease Control and Prevention (CDC), specifically from its Foodborne Disease Surveillance System, which aggregates case reports from 101 designated hospitals across the province.

A total of 57,474 individual foodborne disease case records were initially collected (see Appendix A, Table A1). After excluding cases in which patients received medical treatment outside Zhejiang Province, 56,970 valid cases were retained for analysis. The dataset also includes 91 risk-related indicators, encompassing demographic characteristics, clinical symptoms, dietary exposure, and environmental conditions.

The data cover 11 municipal-level cities in Zhejiang Province, representing a total population of approximately 66 million. This comprehensive coverage ensures the representativeness and reliability of the dataset for identifying temporal trends and regional variations in foodborne disease etiology.

In addition, we incorporated provincial-level socioeconomic and environmental indicators extracted from the Zhejiang Statistical Yearbooks (2014–2024). These indicators were matched with the foodborne disease case records based on corresponding years, enabling integrated analyses of epidemiological trends in relation to regional development factors such as gross domestic product (GDP), agricultural output, population density, and climate-related variables. A detailed list of variables and their descriptive statistics is provided in Appendix A, Table A2.

### 2.2. Data Integration

To enable the application of machine learning models (Random Forest, XGBoost), all collected data were linked and integrated into a unified database. Specifically, the foodborne disease records from Zhejiang Province (a total of 56,970 cases) were expanded by incorporating 91 indicator variables as previously described (see Appendix A, Table A2). This process resulted in a fully integrated dataset comprising 56,970 records. In matching the foodborne disease cases with corresponding regional, climatic, and socioeconomic data, two key variables from the original records, the county (or district) of the patient’s residence and the date of medical consultation, were used as the basis for data alignment.

### 2.3. Data Description

**Figure 1:**
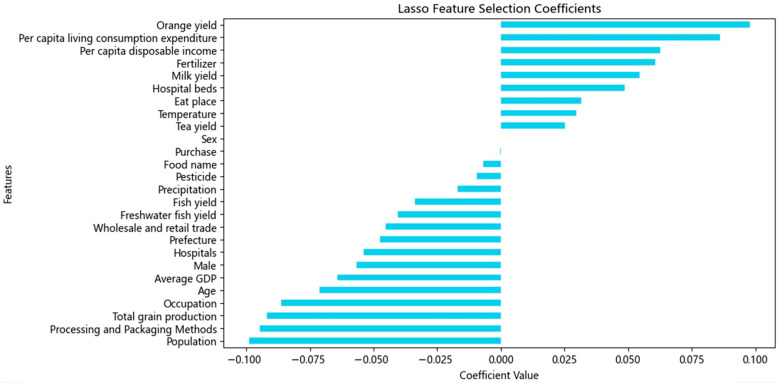
**Lasso feature selection coefficients for predicting foodborne disease categories.** The chart displays the magnitude and direction of influence of each variable based on Lasso regression coefficients, where negative values indicate inverse relationships with foodborne disease types.

To identify key predictors of foodborne disease types, we compiled 91 candidate features based on case records and matched external data sources, including climate, agriculture, demographics, and food-related variables (see Appendix A, Table A2 and Table A3). Lasso regression was applied to perform feature selection using L1 regularization (α = 0.01), resulting in 26 retained features with non-zero coefficients (see Figure 1). These features included ‘Age’, ‘Prefecture’, ‘Food name’, ‘Processing and Packaging Methods’, ‘Occupation’, ‘Purchase’, ‘Eat place’, ‘Climate policy’, ‘Mortality rate’, ‘Fruit yield’, ‘Orange yield’, ‘Cattle’, ‘Hospitals’, ‘Insurance’, and so on. These 26 features were ultimately used as the input variables for subsequent model training.

**Figure 2:**
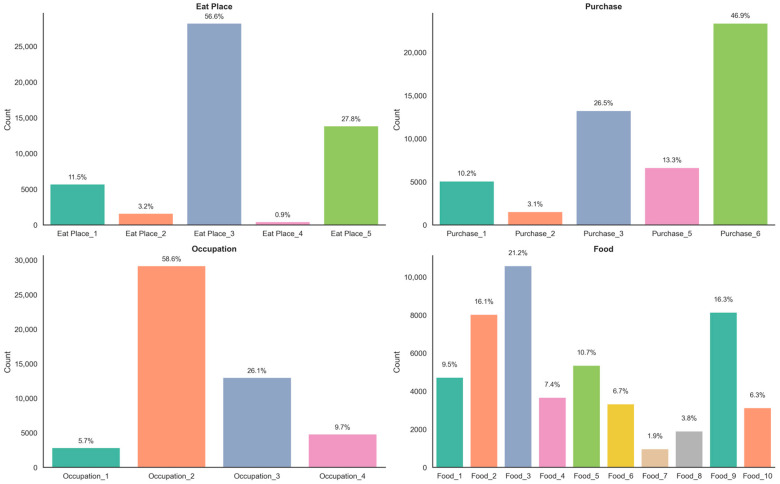
**Distribution of foodborne disease cases by key characteristics in Zhejiang Province, 2014–2023.** This figure presents the distribution of reported foodborne disease cases in Zhejiang Province from 2014 to 2023, classified by four key categorical variables: eating venue (Eat Place), food purchase source (Purchase), patient occupation (Occupation), and implicated food type (Food). Coding schemes for each variable are provided in Appendix A, Table A2.

Descriptive statistical analysis was conducted on the selected variables (see Appendix A, Table A3). To better understand the epidemiological characteristics of foodborne disease cases in Zhejiang Province from 2014 to 2023, we conducted descriptive statistical analysis on key categorical variables, including food category, patient occupation, food purchase source, and type of eating venue (see Figure 2). Among all reported cases, households were the most common eating venues, accounting for 56.6%, followed by other types of locations (27.8%), which mainly include informal or unclassified settings. Catering services accounted for 11.5% of cases, canteens or schools for 3.2%, and retail stores for only 0.9%. These results suggest that home-cooked meals remain a major setting for foodborne disease outbreaks, underscoring the need to strengthen public education on food safety and promote safe food handling practices at the household level. In terms of food procurement sources, the largest proportion of cases (46.9%) were associated with “other” sources, likely referring to unrecorded or informal channels. This was followed by household purchases (26.5%), shops (13.3%), and retail markets (10.2%). Purchases from catering services and canteens each accounted for 3.1%. The high percentage of “other” sources reflects potential limitations in the current data classification system and highlights the need for improved precision in food source reporting. Regarding patient occupation, the majority of cases (58.6%) occurred among migrant workers and related labor groups, followed by individuals from the education and health sectors, including students, teachers, and healthcare workers (26.1%). Patients with unknown or other occupations made up 9.7%, and those employed in the catering industry accounted for 5.7%. This occupational distribution suggests that labor-intensive populations with relatively low health literacy may be at elevated risk of foodborne illness. As for the implicated food categories, aquatic products accounted for the highest proportion (21.2%), followed by meat and meat products (16.1%), nuts/seeds and legumes (16.3%), and grains and grain-based products (9.5%). Vegetables, eggs/dairy, and mixed foods were also significant contributors. Notably, “unknown” or unclassified foods (category 10) accounted for 6.3% of cases, indicating persistent challenges in food traceability and classification accuracy.

In summary, the descriptive analysis reveals that home consumption, informal food sourcing, and labor-intensive occupational groups are the primary correlates of reported foodborne disease cases in Zhejiang Province. These findings provide important insights for risk assessment and the design of targeted public health interventions.

## 3. Methods

To identify key determinants of foodborne disease incidence and improve prediction accuracy, we developed a comprehensive machine learning framework integrating two classifiers, Random Forest and XGBoost, in combination with SMOTE oversampling and Lasso-based feature selection. This approach ensures robust model training, effective handling of imbalanced data, and interpretable identification of significant risk factors.

### 3.1. Machine Learning Algorithms

Random Forest (RF). Random Forest is an ensemble learning algorithm introduced by Breiman [21], designed to improve prediction accuracy and control overfitting by combining the results of multiple decision trees built from bootstrap samples of the data. In RF, each tree is trained on a random subset of the observations and a random subset of the predictors at each split, resulting in diverse models whose aggregated predictions provide enhanced generalization. This approach makes Random Forest particularly robust to noise and outliers, and suitable for high-dimensional data, mixed variable types, and situations with multicollinearity. Additionally, the algorithm naturally provides variable importance measures, enabling interpretation and feature selection.

RF is non-parametric and does not assume any specific distribution of the predictors or outcome variable. It is especially useful in biomedical and epidemiological applications where the relationships between predictors and outcomes are complex, possibly non-linear, and include higher-order interactions. In our study, RF was employed to classify foodborne disease types based on a wide range of demographic, clinical, environmental, and agricultural features [22].

Extreme Gradient Boosting (XGBoost). XGBoost is an advanced implementation of gradient boosting decision trees, developed for speed and performance. Unlike bagging-based methods such as RF, XGBoost is based on boosting, where trees are built sequentially and each new tree corrects the residual errors of the combined previous ensemble. XGBoost introduces several enhancements including L1 and L2 regularization, shrinkage (learning rate), column and row subsampling, and efficient handling of missing data, which together improve both accuracy and generalization. XGBoost has demonstrated state-of-the-art results in many data mining competitions and is particularly effective for tabular datasets with complex feature interactions, class imbalance, or missing values [23].

In the context of multi-class classification problems with potentially imbalanced class distributions and high-dimensional predictors, XGBoost offers improved control over overfitting and often outperforms single-tree or linear models. The algorithm also provides detailed feature importance metrics such as gain, cover, and frequency, facilitating model interpretation and providing insight into key determinants.

Rationale for Algorithm Selection. The selection of Random Forest and XGBoost was motivated by several considerations. First, both algorithms are highly effective for multi-class classification tasks involving structured tabular data, and they are capable of handling both categorical and numerical features without extensive preprocessing. Both perform well even in the presence of missing values and outliers—conditions commonly encountered in real-world public health datasets. Second, their ensemble nature allows them to model complex, non-linear relationships and variable interactions that may not be captured by traditional parametric approaches. Third, both RF and XGBoost offer intrinsic methods for evaluating variable importance, which is critical for interpreting model results and identifying key risk factors of foodborne disease types.

Moreover, the combined use of RF (a bagging-based algorithm that reduces variance) and XGBoost (a boosting-based algorithm that addresses bias and incorporates flexible regularization) enables a comprehensive assessment of model performance, generalizability, and feature relevance. Comparing the results from both models helps identify consistent predictors and provides confidence in the robustness of the findings.

### 3.2. Model Implementation

All data preprocessing, machine learning modeling, and model evaluation were performed using Python 3.8 on the Anaconda 3 Jupyter Notebook 6.4.12 platform. Major libraries included scikit-learn (sklearn), xgboost, pandas, numpy, matplotlib, and seaborn. All analyses were conducted on a personal computer equipped with an Intel i7 processor and 16 GB RAM, running Windows 10.

In this study, model development and evaluation followed these steps: (1) Data preprocessing included automatic encoding detection, removal of rows with missing values, and replacement of infinite values with zero. (2) Feature matrix included all predictors except the target variable (Bacteria), which was encoded as integer classes. (3) The dataset was split into training (80%) and validation (20%) sets using stratified random sampling to maintain class proportions. (4) Both RF and XGBoost models underwent hyperparameter optimization using grid search with five-fold cross-validation. (5) The best models were trained on the entire training set and evaluated on the validation set. (6) Model performance was assessed using accuracy, precision, recall, F1-score, and confusion matrices for each class. (7) Feature importance was extracted and visualized to support epidemiological interpretation.

The dataset exhibited a notable class imbalance, with certain foodborne pathogens represented far less frequently than others. To address this issue, we employed the Synthetic Minority Over-sampling Technique (SMOTE), which generates synthetic samples of the minority class by interpolating between existing observations in feature space. This method has been shown to enhance classifier performance on imbalanced datasets by improving the representation of minority classes during model training. SMOTE was applied exclusively to the training data to avoid information leakage and ensure realistic evaluation on the validation set.

A total of 91 candidate predictors were initially considered, encompassing environmental, behavioral, and socio-economic variables. To reduce dimensionality and prevent overfitting, we applied Lasso regression, a linear model with L1 regularization that shrinks the coefficients of less relevant variables to zero, thereby performing implicit feature selection. The regularization strength (alpha) was set at 0.01 based on the prior literature and tuning experiments. Only features with non-zero coefficients were retained for subsequent modeling, ensuring parsimony and interpretability.

After initial data cleaning and exclusion of out-of-province cases, 56,970 valid records remained, representing all 11 municipal-level cities in Zhejiang Province. The dataset was randomly divided into a training set (80%) and a validation set (20%) to facilitate robust model evaluation. Preprocessing steps included automatic file encoding detection and correction, imputation of missing values using mean or mode strategies, standardization of continuous features and label encoding of categorical variables, and target assignment based on confirmed pathogen categories (e.g., Vibrio parahaemolyticus, Salmonella, Norovirus, E. coli, etc.). These procedures ensured that the input data were clean, consistent, and suitable for training all supervised learning models.

To ensure optimal predictive performance and minimize the risk of overfitting, systematic hyperparameter tuning was conducted for both the Random Forest and XGBoost models in this study. A grid search with five-fold cross-validation was employed to comprehensively evaluate key hyperparameters for each algorithm. For the Random Forest model, the optimal configuration was determined as follows: max_depth = None, max_features = ‘sqrt’, min_samples_leaf = 1, min_samples_split = 10, and n_estimators = 500. This parameter set enables the construction of fully expanded trees, random feature selection at each split, and a substantial number of trees, enhancing the model’s robustness and ability to capture complex patterns. For the XGBoost model, the best hyperparameters were identified as follows: colsample_bytree = 0.8, learning_rate = 0.1, max_depth = 10, n_estimators = 100, and subsample = 0.8. This configuration balances model complexity with generalization capacity and facilitates effective learning from heterogeneous data. The details of the hyperparameter tuning process and the final parameter settings are provided in the Methods Section and are reflected in the reported model performance.

The two classifiers, Random Forest and XGBoost were trained on the selected features using the SMOTE-balanced training set. After training, feature importance was evaluated using both model-specific and model-agnostic approaches. Random Forest provided impurity-based importance scores that reflect each feature’s contribution to classification accuracy, while XGBoost generated gain-based metrics indicating how often and effectively features were used for tree splits. To unify the interpretation across models, SHAP (SHapley Additive exPlanations) was employed to quantify each feature’s marginal contribution to individual predictions, offering a consistent and interpretable assessment of variable influence. Bar plots of SHAP values and model-derived importance scores were created to visually compare the top contributing features across models, enabling a robust evaluation of the most influential risk factors for foodborne disease occurrence.

To comprehensively evaluate model performance, we applied multiple standard classification metrics across both the training and validation sets. These included accuracy, precision, recall, and F1-score, which were derived from classification reports to quantify the models’ ability to correctly identify different foodborne disease categories. Confusion matrices were also generated and visualized using heatmaps, allowing for a clear comparison between predicted and actual labels. Furthermore, we conducted cross-model evaluations to assess the consistency, robustness, and interpretability of predictions among the four classifiers. This comparative analysis not only highlighted the relative strengths and weaknesses of each model but also provided insight into the trade-offs between model complexity, predictive accuracy, and explanatory capacity.

To examine the temporal dynamics of foodborne pathogens, we first conducted exploratory time-series visualizations using monthly and quarterly groupings. Seasonal patterns were then assessed using seasonal differencing and the Augmented Dickey–Fuller test to ensure stationarity. A Seasonal Autoregressive Integrated Moving Average (SARIMA) model was applied to account for trends, seasonality, and randomness in the data. Additionally, classical time-series decomposition was conducted to isolate trend, seasonal, and residual components. Model diagnostics, including the Ljung–Box and heteroscedasticity tests, confirmed the validity and stability of the SARIMA model.

## 4. Results

### 4.1. Trends in Pathogen Composition of Foodborne Diseases in Zhejiang Province

**Figure 3:**
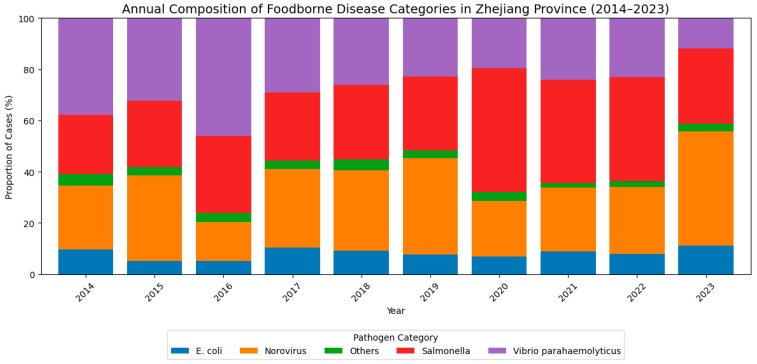
**Annual composition of foodborne disease categories in Zhejiang Province (2014–2023).** Category 1: Salmonella; Category 2: Norovirus; Category 3: Vibrio parahaemolyticus; Category 4: Escherichia coli; Category 0: Other types of bacteria.

Figure 3 illustrates the annual composition of major foodborne disease pathogens in Zhejiang Province from 2014 to 2023, with proportions standardized to 100% each year. The data reveal substantial temporal variation in the dominant causative agents. Vibrio parahaemolyticus and Salmonella were the leading pathogens throughout most of the study period. Vibrio parahaemolyticus showed a marked decline in relative prevalence after 2016, dropping from approximately 45% to below 20% by 2023. In contrast, Salmonella infections increased notably after 2016 and remained the most prominent pathogen from 2019 to 2022, peaking at over 45% in 2020. Norovirus consistently ranked among the top three pathogens, with fluctuations between 20% and 45%, and demonstrated a resurgence in 2023. E. coli infections remained relatively stable and less prevalent, typically accounting for around 5–10% of annual cases. The “Others” category, encompassing less common pathogens, contributed a consistently minor proportion across all years. These temporal trends suggest a shifting landscape of foodborne disease etiology in Zhejiang Province, potentially reflecting changes in environmental conditions, food handling practices, surveillance sensitivity, or public health interventions.

### 4.2. Risk Drivers of Foodborne Disease Types

#### 4.2.1. Model Performance Comparison

Both the Random Forest and XGBoost models were applied to classify foodborne disease types using the selected feature set. Table A4 summarizes the precision, recall, F1-score, and support for each class in both the training and validation sets.

The optimal Random Forest model, determined by five-fold grid search, achieved an overall accuracy of 0.86 on the training set and 0.70 on the validation set. The weighted average F1-score was 0.87 for training and 0.71 for validation (Appendix A, Table A4). The model demonstrated robust performance for the majority classes (e.g., class 1 and class 3), with validation precision and recall of 0.72/0.78 and 0.69/0.66, respectively. However, performance on minority classes remained limited: class 0 and class 4 in the validation set exhibited very low recall (0.00 and 0.21, respectively) and correspondingly low F1-scores. The macro-averaged F1-scores (0.60 for training, 0.38 for validation) highlighted the difficulty of achieving balanced classification across all categories, especially for rare types.

The XGBoost model, following hyperparameter optimization, yielded similar overall results: accuracy of 0.87 on the training set and 0.71 on the validation set. Weighted F1-scores were 0.87 (training) and 0.68 (validation). For the dominant classes, XGBoost achieved high recall (class 1: 0.86, class 3: 0.64) and F1-scores, closely matching the performance of the Random Forest. Minority classes (especially class 0 and class 4) remained challenging to predict, with validation recall of 0.00 and 0.29, respectively. The macro F1-score was 0.40 in the validation set, reflecting the continued imbalance in predictive performance across classes.

Both models exhibited strong performance on the majority foodborne disease types, with validation set accuracy exceeding 0.70 and consistent weighted F1-scores. However, minority classes remained difficult to classify, as evidenced by substantially lower recall and F1-score for these groups. These results underscore the intrinsic challenges of multi-class, imbalanced classification in epidemiological datasets, where the majority of cases cluster in a few categories.

**Figure 4:**
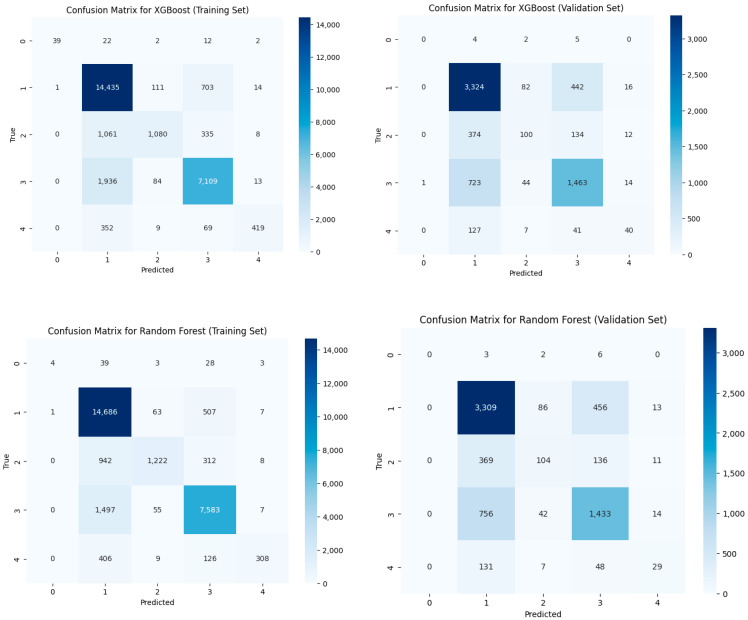
Performance comparison of XGBoost and Random Forest classifiers via confusion matrices on training and validation sets. The Y-axis represents the true class labels (i.e., the actual categories) for the confusion matrix.

Confusion matrix analysis (see Figure 4) revealed that most misclassifications occurred among the minority classes, with substantial confusion between certain underrepresented disease types. Feature importance analysis indicated that demographic, environmental, and food-related variables contributed most to model predictions.

#### 4.2.2. Feature Importance Analysis

**Figure 5:**
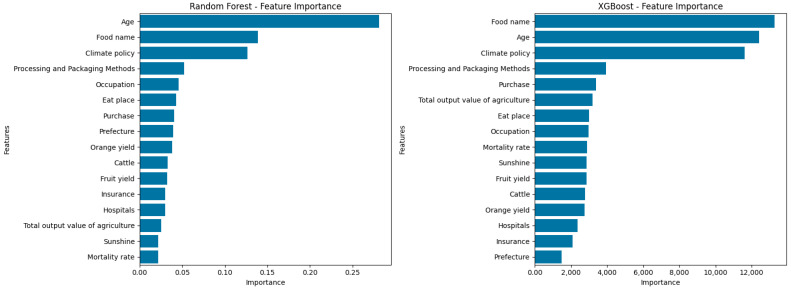
Factors influencing the types of foodborne diseases in Zhejiang Province, China. Feature importance rankings from Random Forest and XGBoost models.

To further elucidate the factors contributing to the classification of foodborne disease types, feature importance was examined for both the Random Forest and XGBoost models (Figure 5).

In the Random Forest model, ‘Age’ was identified as the most influential predictor, followed by ’Food name’ and ‘Climate policy’. Other important features included ‘Processing and Packaging Methods’, ‘Occupation’, ‘Eat place’, and several agricultural or environmental variables such as ‘Orange yield’, ‘Fruit yield’, and ’Mortality rate’. The prominence of age- and food-related characteristics highlights the combined effect of host susceptibility and exposure routes in foodborne disease risk, while the significance of climate and agricultural variables suggests the importance of external and environmental factors.

In the XGBoost model, ‘Food name’ and ‘Age’ ranked as the top two most important features, followed by ‘Climate policy’, ‘Processing and Packaging Methods’, and ‘Purchase’. Notably, both models consistently identified food attributes, age, and policy/environmental factors as primary determinants of disease type. The overlap in high-ranking variables between the two models underscores the robustness of these predictors across different machine learning frameworks.

These results indicate that both individual level (e.g., age, occupation) and environmental or systemic factors (e.g., climate policy, agricultural production) jointly shape the distribution of foodborne disease types. The findings support the multifactorial nature of foodborne disease risk and provide a basis for targeted interventions and further etiological investigation.

### 4.3. Time Pattern Analysis of Foodborne Disease Types

**Figure 6:**
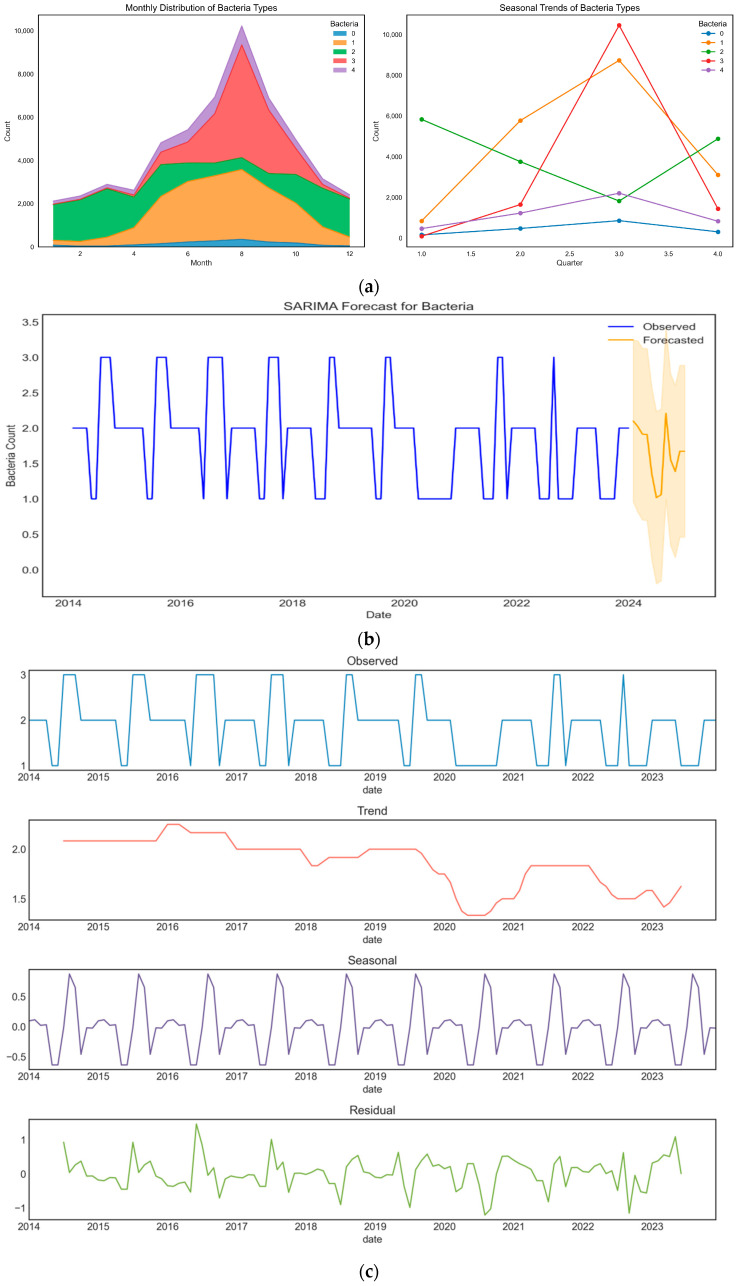
**Time pattern of foodborne disease types.** (**a**) Distribution of different bacterial types in foodborne diseases across months. Category 1: Salmonella; Category 2: Norovirus; Category 3: Vibrio parahaemolyticus; Category 4: Escherichia coli; Category 0: Other types of bacteria. (**b**) Temporal patterns of different bacterial types. (**c**) Decomposition of the time series of foodborne disease counts. The Y-axis indicates “Standardized Bacteria Count”. The four subplots represent the observed series (Observed), long-term trend (Trend), seasonal component (Seasonal), and random residual (Residual), respectively. Both trend and seasonal components have been standardized for comparative purposes.

Through the analysis of the temporal patterns of bacterial types, the study found significant seasonal differences in the transmission of various bacterial types. To investigate the seasonal variation in bacterial types, the data were first grouped by month, and an area plot was used to illustrate the incidence rate of each bacterial type across different months. Additionally, quarterly grouping was applied, and a line graph was used to display the variation in the incidence frequency of each bacterial type across the four seasons (Figure 6a). Norovirus (Category 2) showed higher incidence rates in the spring and winter, suggesting that climatic conditions during the warmer seasons may facilitate the transmission of these pathogens. Vibrio parahaemolyticus (Category 3) demonstrates strong seasonality, with significantly elevated incidence during the warm summer months. This pattern is consistent with its known ecological preference for high-temperature and high-humidity conditions, which facilitate bacterial growth and increase the risk of food contamination. In contrast, Salmonella (Category 1) and other bacterial types (Category 0) did not exhibit significant seasonal variations, indicating that their transmission mechanisms may not be significantly influenced by seasonal changes. For Escherichia coli (Category 4), which shows a marked increase in incidence during the summer months. This trend may be attributed to the bacteria’s preference for warmer temperatures, which promote its growth and survival in food sources. Escherichia coli, a pathogen commonly associated with foodborne outbreaks, thrives under these conditions, thus increasing the risk of contamination. Unlike Vibrio parahaemolyticus (Category 3), which has a strong seasonality linked to high temperature and humidity, Escherichia coli’s peak during the warmer months reflects its adaptive survival strategy in temperature-sensitive environments.

In addition, to further elucidate the temporal regularity and short-term trends of foodborne disease incidence, a Seasonal Autoregressive Integrated Moving Average (SARIMA) model was constructed using historical surveillance data to forecast pathogen occurrence over the upcoming period (see Figure 6b and Appendix A, Table A5). The SARIMA model effectively captured the seasonal fluctuations observed in previous years and provided reasonable interval predictions for the projected bacterial counts in 2023–2024. The relatively wide prediction intervals indicate that future disease incidence may be influenced by a variety of unpredictable factors, such as extreme weather events or changes in human behavior. Therefore, dynamic surveillance and real-time early warning based on time series models such as SARIMA are essential for strengthening risk management and preparedness for foodborne diseases.

We further investigated the temporal patterns of different bacterial types. Through seasonal differencing and the ADF stationarity test (ADF Statistic: −4.718; *p*-value: 0.000), we confirmed the stationarity of the foodborne disease bacterial type data after seasonal adjustment. The Seasonal Autoregressive Integrated Moving Average (SARIMA) model was employed to capture trends, seasonal fluctuations, and randomness in the foodborne disease time series data. The impact of seasonal factors on bacterial type variation was significant, particularly the coefficient and significance level of the seasonal moving average term (MA.S.L12), suggesting that seasonal changes, such as temperature and humidity, may play a crucial role in bacterial transmission. The model fit was good, and although there were some non-normal residuals, they did not significantly affect the model’s predictive capability. The Ljung–Box and heteroscedasticity tests supported the model’s validity and stability.

The time effect decomposition revealed the temporal patterns of bacterial types, with the fluctuation of seasonal components being particularly prominent. Figure 6c presents the results of the time effect decomposition of bacterial type data, consisting of four subplots: the original observed values, trend component, seasonal component, and residual component. It is evident that the data exhibit clear periodic fluctuations, with repeated oscillation patterns across different years. The trend plot demonstrates the long-term change trend of bacterial types, showing that the average level of bacterial types remained relatively stable between 2014 and 2023, with no obvious upward or downward trend. The seasonal component plot reflects the peaks and valleys of bacterial types within fixed time periods each year, indicating that the variation in bacterial types is significantly influenced by seasonal factors. The residual plot suggests that, although most of the variation can be explained by the trend and seasonal components, there are still some fluctuations not captured by the model. These fluctuations may represent randomness or other unconsidered factors (see Figure 6c).

## 5. Conclusions

This study provides a comprehensive assessment of the temporal evolution, risk determinants, and seasonal dynamics of foodborne disease pathogens in Zhejiang Province from 2014 to 2023. The analysis revealed pronounced shifts in the dominant etiological agents over the past decade: The relative prevalence of Vibrio parahaemolyticus declined markedly after 2016, while Salmonella emerged as the leading pathogen from 2019 onwards, peaking in 2020. Norovirus consistently ranked among the top three pathogens, exhibiting notable fluctuations and a resurgence in 2023. Escherichia coli infections remained relatively stable and less prevalent, and other bacterial types contributed a consistently minor proportion each year. These findings suggest that changes in environmental conditions, food handling practices, surveillance sensitivity, and public health interventions have collectively shaped the landscape of foodborne disease etiology in the region.

In terms of risk prediction, both Random Forest and XGBoost models achieved robust performance in classifying the major foodborne disease types, with validation set accuracies exceeding 0.70 and strong weighted F1-scores. Feature importance analyses highlighted patient age, food name, climate policy, and processing methods as key determinants, underscoring the joint influence of host susceptibility, exposure routes, and environmental or systemic factors on disease type distribution. However, the models’ ability to identify minority classes remained limited, reflecting the inherent challenges of multi-class, imbalanced classification in epidemiological datasets and signaling the need for further methodological refinement.

Temporal analyses demonstrated significant seasonal fluctuations in the incidence of specific bacterial types, particularly Vibrio parahaemolyticus and Norovirus, which showed peaks during the warmer months. The SARIMA model effectively captured these seasonal variations and provided dynamic forecasts of future pathogen occurrence, while time series decomposition confirmed the predominance of periodic and trend components in the temporal patterns of foodborne diseases. Nevertheless, some residual variation remained unexplained, likely due to stochastic or unmeasured influences.

Overall, this study confirms the multifactorial and spatiotemporally heterogeneous nature of foodborne disease risk, offering theoretical and practical insights for dynamic early warning, targeted interventions, and evidence-based policy development. Future work should prioritize the integration of high-resolution individual-level data, multi-source information fusion, and the advancement of methods for rare class detection, with the aim of improving model generalizability and strengthening public health preparedness.

## 6. Discussion

This study provides a comprehensive investigation into the temporal and structural dynamics of foodborne diseases in Zhejiang Province from 2014 to 2023, yielding insights into changing pathogen prevalence, key predictors, and seasonal transmission patterns. These findings contribute to the broader public health effort to achieve Sustainable Development Goal 3 (SDG 3: Good Health and Well-being) by enhancing disease surveillance and prevention systems.

First, the longitudinal analysis of pathogen composition revealed a shifting landscape in foodborne disease etiology. The declining dominance of Vibrio parahaemolyticus and the concurrent rise of Salmonella suggest evolving environmental or behavioral risk contexts. These changes may be attributed to improvements in seafood hygiene practices, modifications in dietary habits, or shifts in public health reporting accuracy. Notably, Norovirus maintained a consistently high prevalence and resurged in 2023, underscoring the need for persistent vigilance in viral gastroenteritis control. These findings align with the WHO’s emphasis on food safety as a fundamental component of health security under SDG 3.9, which targets reductions in deaths and illnesses from hazardous food and water.

Second, machine learning models demonstrated that a relatively small subset of well-selected variables can effectively predict the causative agent of foodborne diseases. The high classification accuracy achieved by Random Forest (86%) and XGBoost (87%) models confirms the predictive value of features both individual-level (e.g., age, occupation) and environmental or systemic factors (e.g., climate policy, agricultural production). These predictors are not only statistically robust but also actionable from a policy perspective. For instance, food names and processing methods can guide targeted inspections and traceability interventions, while demographic indicators like age highlight vulnerable population groups requiring enhanced protection.

Third, the seasonal analysis emphasized the climatic sensitivity of specific pathogens. Vibrio parahaemolyticus and Norovirus showed strong seasonal patterns, peaking in warmer seasons, consistent with the prior microbiological and epidemiological literature. The SARIMA modeling and time decomposition further validated the presence of significant seasonal components, implying that disease prevention strategies must integrate climate-based early warning systems. These insights are particularly relevant to SDG 13 (Climate Action) and SDG 3.D, which call for improved capacity for early warning and risk reduction in national health risks.

However, some limitations should be acknowledged. First, while the dataset is large and comprehensive, it reflects only reported cases from sentinel hospitals, potentially omitting subclinical or underreported infections. Second, the use of administrative-level aggregated features (e.g., climate, agriculture) may mask individual-level variation. Future research could benefit from higher-resolution data, including household-level exposures and microbiological subtyping, to enhance causal inference and generalizability.

Furthermore, it is important to further clarify that while LASSO regression was used for preliminary feature selection, we fully recognize its inherent limitations as a linear method—particularly in datasets where complex non-linear relationships may exist. To mitigate potential information loss, we systematically incorporated Random Forest and XGBoost in the subsequent modeling stages, both of which are capable of capturing high-order, non-linear effects and interactions. Additionally, we conducted model-based feature importance ranking using these algorithms to re-evaluate the contribution of all retained variables. Our results demonstrate that certain features, such as food type and climate policy, were consistently assigned high importance by non-linear models, even after the initial LASSO selection, highlighting the models’ capacity to identify complex interaction and non-linear signals. The stability of feature importance rankings across both Random Forest and XGBoost further supports the robustness of the key predictors identified in this study. This multi-stage process of feature selection and model interpretation effectively balances dimensionality reduction with information retention and enhances model generalizability across different data structures. Future work could further explore the integration of multiple feature selection strategies, feature engineering, and interaction term expansion to better address highly non-linear and imbalanced epidemiological datasets.

This study highlights the value of integrating machine learning, environmental data, and time-series modeling in understanding foodborne disease dynamics. It underscores the need for targeted, seasonally adaptive, and geographically sensitive public health strategies to reduce the burden of foodborne illnesses and advance sustainable health system resilience.

Future research should integrate higher-resolution data sources, such as individual and household-level exposure records, dietary behavior, and pathogen subtyping, to address the limitations of aggregated datasets. This approach would enable more precise identification of high-risk populations and critical transmission routes, thereby enhancing the targeting and scientific basis of disease prevention efforts. Additionally, the application of spatial epidemiology and advanced artificial intelligence modeling is encouraged to systematically assess the roles of geographic heterogeneity, spatial clustering, and climate change in foodborne disease dynamics, providing a more forward-looking foundation for early warning and adaptive intervention.

Moreover, future work should emphasize the integration of multi-source big data—including electronic health records, food traceability systems, and remote sensing meteorological data—with state-of-the-art algorithms such as deep learning and graph neural networks. It is recommended that future research employ causal inference and policy simulation methods to evaluate the real-world effectiveness and cost-effectiveness of different public health interventions. Additionally, strengthening international comparisons and cooperation is essential to advance knowledge sharing and global governance in foodborne disease prevention.

## Data Availability

The original contributions presented in the study are included in the article. For access to the anonymized dataset and the Python code used in this study, please contact the corresponding author.

## Appendix A

**Table A1:** Example case records of foodborne diseases in Zhejiang Province.

Code	Items	Contents
1	Case ID	***
2	Outpatient Number	***
3	Follow-up Visit (Yes/No)	No
4	Hospitalized (Yes/No)	No
5	Inpatient Number	***
6	Gender	Female
7	Date of Birth	1999-01-01
8	Age	26
9	Occupation	Healthcare workers
10	Workplace	***
11	Patient Category	Other districts in this city
12	Address—Province	Zhejiang
13	Address—City	Ningbo
14	Address—District/County	Haishu
15	Detailed Address	***
16	Onset Date	2023-02-16 02
17	Consultation Date	2023-02-16 16
18	Time of Death	-
19	Main Symptoms and Signs	[Digestive system] Nausea, vomiting once/day
20	Medical History	None
21	Preliminary Diagnosis	[Viral] Norovirus
22	Antibiotic Use Before Consultation (Yes/No)	No
23	Suspected Foodborne Case (Yes/No)	Yes
24	Biological Sample (Yes/No)	Yes
25	Food Name	beef noodles
26	Food Category	Meat and meat products
27	Processing and Packaging Method	Catering service industry
28	Purchase Province	Zhejiang
29	Purchase City	Ningbo
30	Purchase District/County	Jiangbei
31	Detailed Purchase Address	***
32	Purchase Location Type	Shop
33	Eating Location—Province	Zhejiang
34	Eating Location—City	Ningbo
35	Eating Location—District/County	Jiangbei
36	Detailed Eating Location Address	***
37	Eating Location Type	Catering
38	Number of People Dining Together	1
39	Eating Time	2023-02-10 19
40	Food Sample Collected (Yes/No)	No
41	Other People Affected (Yes/No)	No
42	Sample ID	***
43	Sample Type	Fecal sample
44	Sample Value	5
45	Sample Unit	Pcs
46	Sample Collection Date	2023-02-13
47	Test Item	Norovirus
48	Testing Institution	***
49	Testing Date	2023-02-13
50	Qualitative Result	+
51	Testing Unit	
52	Strain Isolated (Yes/No)	Yes
53	Strain ID	***
54	Identification Method	Nomal PCR
55	Target Gene Detection	-
56	Serotyping	-
57	Identification Conclusion	Norovirus
91	Strain Depository Organization	***

Note: For patient privacy, this table contains fictitious data modeled after real information. *** indicates redacted personal information. +: Positive. -: Not available.

**Table A2:** Descriptions of the identified factors, their units, and data sources.

Code	Variables	Description	Data Cleaning Rules	Data Sources
1	Prefecture	The patient’s municipal address	Encoded with the corresponding administrative division codes, for example: Hangzhou → 3301; Nanjing → 3201; Wuhan → 4201.	Zhejiang Provincial Center for Disease Control and Prevention, 2014–2023.
2	Food	Food category	The encoding for food categories is as follows: Grains and their products (including starch sugars, baked goods, and various staple foods) = 1; Meat and meat products = 2; Oils and fats = 2; Aquatic animals and their products = 3; Eggs and egg products = 4; Dairy products = 4; Fruits and their products (including dried and preserved fruits) = 5; Vegetables and their products = 6; Fungi and their products = 6; Algae and their products = 6; Legumes and their products = 7; Nuts and seeds and their products = 7; Beverages and frozen drinks = 8; Alcoholic beverages and their products = 8; Packaged drinking water (including bottled water) = 8; Mixed foods = 9; Various foods = 9; Blank = 10; Packaged bulk products = 10; Unknown foods = 10; Condiments = 10; Other foods = 10; Candies, chocolates, honey, and their products = 10; Infant foods = 10	
3	Age	The patient’s age, which includes different units such as “years,” “months,” and “days.”	The age values are standardized and converted into numerical values expressed in years. For example: “40 years old” → 40; “1 year and 4 months” → 1 + 4/12 ≈ 1.33; “8 months” → 8/12 ≈ 0.67; “9 months and 30 days” → 9/12 + 30/365 ≈ 0.78	
4	Sex	Record the patient’s gender.	Convert to numerical values: Male = 1; Female = 0.	
5	Occupation	Indicating the occupation of the patient	The coding for numerical values is as follows: Catering industry = 1; Migrant workers = 2; Unknown = 4; Teachers = 3; Students = 3; Others = 4; Farmers = 2; Dispersed children = 2; Commercial services = 1; Pastoralists = 2; Fishermen = 2; Administrative staff = 3; Preschool children = 3; Healthcare workers = 3; Retired personnel = 3; Homemakers and unemployed = 2; Workers = 2.	
6	Eat place	The type of location where food is consumed.	The categorical variables were encoded as numerical values: Catering industry = 1; Canteen = 2; Household = 3; Rural banquets = 1; Retail market = 4; Other = 5; School = 2; Type of eating venue = 5.	
7	Purchase	Represents the type of location where food is purchased.	The categorical variables were encoded as follows: Catering Industry = 1, Canteen = 2, Household = 3, Street Vendor = 1, Retail Market = 4, Others = 6, and Shop = 5.	
8	Bacteria	Indicating the bacterial classification or category.	Classification codes: Salmonella = 1; Norovirus = 2; Vibrio parahaemolyticus = 3; Escherichia coli = 4; Others =0	
9	Diagnosis	The duration of “time of consultation” and “time of onset,” measured in hours.	The “time of visit” minus the “time of onset” yields the duration of medical consultation in hours.	
10	GDP	Gross Domestic Product	Gross Domestic Product in prefectures of Zhejiang province during 2014–2023. Unit: billion yuan	
11	GDP1	Gross Domestic Product of the Primary Industry	Gross Domestic Product of the Primary Industry in prefectures of Zhejiang province during 2014–2023. Unit: billion yuan	
12	GDP2	Gross Domestic Product of the Secondary Industry	Gross Domestic Product of the Secondary Industry in prefectures of Zhejiang province during 2014–2023. Unit: billion yuan	
13	GDP3	Gross Domestic Product of the Tertiary Industry	Gross Domestic Product of the Tertiary Industry in prefectures of Zhejiang province during 2014–2023. Unit: billion yuan	
14	Average GDP	Gross Domestic Product per capita	Gross Domestic Product per capita in prefectures of Zhejiang province during 2014–2023. Unit: yuan	
15	Household	Total Number of Households	Total Number of Households in prefectures of Zhejiang province during 2014–2023. Unit: household	
16	Population	Total Population	Total Population in prefectures of Zhejiang province during 2014–2023. Unit: ten thousand people	
17	Mortality	Mortality rate	Mortality rate in prefectures of Zhejiang province during 2014–2023. Unit: ‰	
18	Employment	Number of Employed Persons	Number of Employed Persons in the Entire Society at the End of the Year in prefectures of Zhejiang province during 2014–2023. Unit: Ten Thousand Persons	
19	Income disposable	The per capita disposable income of urban and rural residents	The per capita disposable income of urban and rural residents in prefectures of Zhejiang province during 2014–2023. Unit: yuan	
20	Consumption expenditure	The per capita living consumption expenditure	The per capita living consumption expenditure of urban and rural residents in prefectures of Zhejiang province during 2014–2023. Unit: yuan	
21	Total agriculture	The total output value of agriculture	The total output value of agriculture, forestry, animal husbandry, and fishery in prefectures of Zhejiang province during 2014–2023. Unit: billion yuan	
22	Sown area	The area of crop planting	The area of crop planting in prefectures of Zhejiang province during 2014–2023. Unit: thousand hectares	
23	Total grain yield	Total Grain yield	Total Grain yield in prefectures of Zhejiang province during 2014–2023. Unit: million tons	
24	Cereal yield	Cereal yield	Cereal yield in prefectures of Zhejiang province during 2014–2023. Unit: tons	Zhejiang Statistical Yearbook
25	Rapeseed yield	yield of rapeseed	Yield of rapeseed in prefectures of Zhejiang province during 2014–2023. Unit: ten thousand tons	
26	Cotton yield	Cotton yield	Cotton yield in prefectures of Zhejiang province during 2014–2023. Unit: tons	
27	Fruit yield	Fruit yield	Fruit yield in prefectures of Zhejiang province during 2014–2023. Unit: tons	
28	Meat yield	Meat yield	Meat yield in prefectures of Zhejiang province during 2014–2023. Unit: tons	
29	Pork yield	Pork yield	Pork yield in prefectures of Zhejiang province during 2014–2023. Unit: tons	
30	Egg yield	Egg yield	Egg yield in prefectures of Zhejiang province during 2014–2023. Unit: tons	
31	Milk yield	Milk yield	Milk yield in prefectures of Zhejiang province during 2014–2023. Unit: tons	
32	Fish yield	Aquatic product yield	Aquatic product yield in prefectures of Zhejiang province during 2014–2023. Unit: ten thousand tons	
33	Marine fish yield	Marine fish yield	Marine fish yield in prefectures of Zhejiang province during 2014–2023. Unit: ten thousand tons	
34	Freshwater fish yield	Freshwater fish yield	Freshwater fish yield in prefectures of Zhejiang province during 2014–2023. Unit: Ten Thousand tons	
35	agricultural plastic	The usage of agricultural plastic film	The usage of agricultural plastic film in prefectures of Zhejiang province during 2014–2023. Unit: tons	
36	Fertilizer	Usage of Agricultural Fertilizers	Usage of Agricultural Fertilizers in prefectures of Zhejiang province during 2014–2023. Unit: tons	
37	Nitrogen	Nitrogen fertilizer usage	Nitrogen fertilizer usage in prefectures of Zhejiang province during 2014–2023. Unit: tons	
38	Phosphate	Phosphate fertilizer usage	Phosphate fertilizer usage in prefectures of Zhejiang province during 2014–2023. Unit: tons	
39	Potassium	Potassium fertilizer application	Potassium fertilizer application in prefectures of Zhejiang province during 2014–2023. Unit: tons	
40	Compound	Compound fertilizer usage	Compound fertilizer usage in prefectures of Zhejiang province during 2014–2023. Unit: tons	
41	Pesticide	Pesticide Usage	Pesticide Usage in prefectures of Zhejiang province during 2014–2023. Unit: tons	
42	Wholesale retail	Total Sales of Goods in Wholesale and Retail Trade Above Designated Size	Total Sales of Goods in Wholesale and Retail Trade Above Designated Size in prefectures of Zhejiang province during 2014–2023. Unit: hundred million yuan	
43	Income	Total Fiscal Revenue	Total Fiscal Revenue in prefectures of Zhejiang province during 2014–2023. Unit: hundred million yuan	Zhejiang Statistical Yearbook
44	Expenditure	Local Government Fiscal Expenditure	Local Government Fiscal Expenditure in Zhejiang province during 2014–2023. Unit: hundred million yuan	
45	Public expenditure	General Public Service Expenditure	General Public Service Expenditure in prefectures of Zhejiang province during 2014–2023. Unit: hundred million yuan	
46	Temperature	Annual Average Temperature	Annual Average Temperature in prefectures of Zhejiang province during 2014–2023. Unit: 0.1 °C	
47	Temperature1	Average Temperature in January	Average Temperature in January in prefectures of Zhejiang province during 2014–2023. Unit: Celsius	
48	Temperature2	Average Temperature in February	Average Temperature in February in prefectures of Zhejiang province during 2014–2023. Unit: Celsius	
49	Temperature3	Average Temperature in March	Average Temperature in March in prefectures of Zhejiang province during 2014–2023. Unit: Celsius	
50	Temperature4	Average Temperature in April	Average Temperature in April in prefectures of Zhejiang province during 2014–2023. Unit: Celsius	
51	Temperature5	Average Temperature in May	Average Temperature in May in prefectures of Zhejiang province during 2014–2023. Unit: Celsius	
52	Temperature6	Average Temperature in June	Average Temperature in June in prefectures of Zhejiang province during 2014–2023. Unit: Celsius	
53	Temperature7	Average Temperature in July	Average Temperature in July in prefectures of Zhejiang province during 2014–2023. Unit: Celsius	
54	Temperature8	Average Temperature in August	Average Temperature in August in prefectures of Zhejiang province during 2014–2023. Unit: Celsius	
55	Temperature9	Average Temperature in September	Average Temperature in September in Zhejiang province during 2014–2023. Unit: Celsius	
56	Temperature10	Average Temperature in October	Average Temperature in October in prefectures of Zhejiang province during 2014–2023. Unit: Celsius	
57	Temperature11	Average Temperature in November	Average Temperature in November in prefectures of Zhejiang province during 2014–2023. Unit: Celsius	
58	Temperature12	Average Temperature in December	Average Temperature in December in prefectures of Zhejiang province during 2014–2023. Unit: Celsius	
59	Precipitation	Annual Precipitation	Annual Precipitation in prefectures of Zhejiang province during 2014–2023. Unit: 0.1 mm	
60	Precipitation1	Precipitation in January	Precipitation in January in prefectures of Zhejiang province during 2014–2023. Unit:0.1 mm	
61	Precipitation2	Precipitation in February	Precipitation in February in prefectures of Zhejiang province during 2014–2023. Unit: 0.1 mm	
62	Precipitation3	Precipitation in March	Precipitation in March in prefectures of Zhejiang province during 2014–2023. Unit: 0.1 mm	
63	Precipitation4	Precipitation in April	Precipitation in April in prefectures of Zhejiang province during 2014–2023. Unit: 0.1 mm	
64	Precipitation5	Precipitation in May	Precipitation in May in prefectures of Zhejiang province during 2014–2023. Unit: 0.1 mm	
65	Precipitation6	Precipitation in June	Precipitation in June in prefectures of Zhejiang province during 2014–2023. Unit: 0.1 mm	
66	Precipitation7	Precipitation in July	Precipitation in July in prefectures of Zhejiang province during 2014–2023. Unit: 0.1 mm	
67	Precipitation8	Precipitation in August	Precipitation in August in prefectures of Zhejiang province during 2014–2023. Unit: 0.1 mm	
68	Precipitation9	Precipitation in September	Precipitation in September in prefectures of Zhejiang province during 2014–2023. Unit: 0.1 mm	
69	Precipitation10	Precipitation in October	Precipitation in October in prefectures of Zhejiang province during 2014–2023. Unit: 0.1 mm	
70	Precipitation11	Precipitation in November	Precipitation in November in prefectures of Zhejiang province during 2014–2023. Unit: 0.1 mm	
71	Precipitation12	Precipitation in December	Precipitation in December in prefectures of Zhejiang province during 2014–2023. Unit: 0.1 mm	
72	Sunshine	Total Annual Sunshine Hours	Total Annual Sunshine Hours in prefectures of Zhejiang province during 2014–2023. Unit: 0.1 h	
73	Sunshine1	Sunshine Hours in January	Sunshine Hours in January in prefectures of Zhejiang province during 2014–2023. Unit: 0.1 h	Zhejiang Statistical Yearbook
74	Sunshine2	Sunshine Hours in February	Sunshine Hours in February in prefectures of Zhejiang province during 2014–2023. Unit: 0.1 h	
75	Sunshine3	Sunshine Hours in March	Sunshine Hours in March in prefectures of Zhejiang province during 2014–2023. Unit: 0.1 h	
76	Sunshine4	Sunshine Hours in April	Sunshine Hours in April in prefectures of Zhejiang province during 2014–2023. Unit: 0.1 h	
77	Sunshine5	Sunshine Hours in May	Sunshine Hours in May in prefectures of Zhejiang province during 2014–2023. Unit: 0.1 h	
78	Sunshine6	Sunshine Hours in June	Sunshine Hours in June in prefectures of Zhejiang province during 2014–2023. Unit: 0.1 h	
79	Sunshine7	Sunshine Hours in July	Sunshine Hours in July in prefectures of Zhejiang province during 2014–2023. Unit: 0.1 h	
80	Sunshine8	Sunshine Hours in August	Sunshine Hours in August in prefectures of Zhejiang province during 2014–2023.Unit: 0.1 h	
81	Sunshine9	Sunshine Hours in September	Sunshine Hours in September in prefectures of Zhejiang province during 2014–2023. Unit: 0.1 h	
82	Sunshine10	Sunshine Hours in October	Sunshine Hours in October in prefectures of Zhejiang province during 2014–2023. Unit: 0.1 h	
83	Sunshine11	Sunshine Hours in November	Sunshine Hours in November in prefectures of Zhejiang province during 2014–2023. Unit: 0.1 h	
84	Sunshine12	Sunshine Hours in December	Sunshine Hours in December in prefectures of Zhejiang province during 2014–2023. Unit: 0.1 h	
85	water resources	Total Water Resources	Total Water Resources in prefectures of Zhejiang province during 2014–2023. Unit: hundred million cubic meters	
86	water supply	Total Water Supply	Total Water Supply in prefectures of Zhejiang province during 2014–2023. Unit: hundred million cubic meters	
87	Hospitals	Number of Hospitals and Health Centers	Number of Hospitals and Health Centers in prefectures of Zhejiang province during 2014–2023 (Units).	
88	Hospital beds	Number of Hospital Beds	Number of Hospital Beds in prefectures of Zhejiang province during 2014–2023 (Units).	
89	Doctors	Number of Doctors	Number of doctors in prefectures of Zhejiang province during 2014–2023 (Units).	
90	Insurance	Number of Basic Medical Insurance Enrollees	Number of Basic Medical Insurance Enrollees in prefectures of Zhejiang province during 2014–2023. Unit: ten thousand yuan	Zhejiang Statistical Yearbook
91	Climate policy	Climate Policy Index	Using manual auditing and the deep learning algorithm MacBERT model, we constructed the CCPU index for China at the national, provincial, and major city levels from January 2000 to December 2022. This index is based on 1,755,826 articles from six mainstream newspapers in China: People’s Daily, Guangming Daily, Economic Daily, Global Times, Science and Technology Daily, and China News Service. The research framework consists of six parts: data collection, data cleaning, manual auditing, model construction, index calculation and normalization, and technical validation.	[24]

**Table A3:** Data description.

Code	Variable	Count	Mean	Std	Min	Max
1	age	56,970	32.11	22.00	0	99
2	sex	56,970	0.55	0.50	0	1
3	occupation	56,970	2.41	0.74	1	4
4	food	51,607	4.82	2.91	1	10
5	purchase	49,570	4.44	1.77	1	6
6	eat place	51,468	3.33	1.24	1	5
7	bacteria	56,970	3.25	2.00	1	6
8	diagnosis	56,970	27.45	41.32	0	2221
9	City code	56,970	3305.84	3.31	3301	3311
10	year	56,970	2019.34	2.67	2014	2023
11	GDP	42,749	6181.53	4608.54	971.47	18,753
12	GDP1	42,749	193.87	92.02	21.05	382
13	GDP2	42,749	2608.88	1731.65	369.93	7413
14	GDP3	42,749	3112.68	2561.17	457.63	12,287.31
15	average GDP	42,749	98,051.57	31,005.40	39,721	167,134
16	household	48,937	1,515,721.00	783,651.17	339,224	2,664,533
17	population	48,444	452.29	253.31	76.66	846.75
18	mortality	41,061	5.92	0.79	4.5	8
19	employment	42,749	385.57	201.08	72.14	759.68
20	Income disposable	41,332	48,961.18	11,069.14	22,426	70,281
21	consumption expenditure	41,332	31,268.95	7043.39	13,875	46,440
22	total agriculture	42,749	310.34	145.54	30.29	589.82
23	sown area	42,749	201.09	67.76	13.45	320.18
24	total grain yield	44,594	56.25	24.41	2.48	122.34
25	cereal yield	42,749	514,214.28	213,044.53	16,832	1,158,805
26	rapeseed yield	43,252	2.33	1.77	0.13	6.62
27	cotton yield	40,022	880.62	1323.54	1	9488
28	fruit yield	42,749	699,250.71	397,609.49	63,002	1,499,253
29	meat yield	42,749	107,953.67	56,592.71	4686	322,988
30	pork yield	42,749	74,989.71	50,859.05	1524	250,047
31	egg yield	42,749	36,035.15	24,953.02	189	130,870
32	milk yield	40,379	17,794.60	16,598.84	2	65,073
33	freshwater fish yield	43,252	12.15	13.27	0.06	60.46
34	agricultural plastic	43,252	6505.72	3589.66	325	13,567
35	fertilizer	42,749	70,619.17	25,618.09	3907	112,811
36	nitrogen	43,252	31,332.97	16,029.53	1500	78,100
37	phosphate	43,854	6618.99	3886.90	0	14,700
38	potassium	43,252	5428.94	2510.99	100	12,500
39	compound	43,252	26,427.04	14,814.84	1300	56,000
40	pesticide	43,252	3841.39	1705.31	324	7510
41	wholesale retail	42,464	8950.83	11,985.53	312.34	46,799.786
42	income	42,464	1189.07	1186.74	119.02	4590.08
43	expenditure	42,464	863.83	595.26	72.23	2542.09
44	public expenditure	42,464	86.45	49.71	11.12	210.01
45	temperature1	41,332	73.22	17.02	35	115
46	temperature2	41,332	85.26	22.42	49	137
47	temperature3	41,332	131.29	12.83	105	160
48	temperature4	41,332	177.92	10.35	148	204
49	temperature5	41,332	222.94	13.27	190	254
50	temperature6	41,332	254.28	10.57	226	280
51	temperature7	41,332	292.79	15.19	256	329
52	temperature8	41,332	294.70	13.20	259	334
53	temperature9	41,332	255.47	12.77	232	286
54	temperature10	41,332	202.73	12.30	180	234
55	temperature11	41,332	151.80	14.48	126	184
56	temperature12	41,332	87.19	18.41	48	134
57	temperature	41,332	186.07	7.73	168	200
58	precipitation1	41,332	795.06	500.62	55	2153
59	precipitation2	41,332	897.30	538.90	169	2764
60	precipitation3	41,332	1376.98	522.21	467	2879
61	precipitation4	41,332	1187.64	602.80	369	3576
62	precipitation5	41,332	1746.45	820.46	523	4241
63	precipitation6	41,332	2764.66	995.91	877	5882
64	precipitation7	41,332	1894.35	1338.43	208	6353
65	precipitation8	41,332	2018.23	1231.23	223	4990
66	precipitation9	41,332	1745.05	1166.36	62	6069
67	precipitation10	41,332	823.14	780.02	30	3577
68	precipitation11	41,332	845.91	531.14	57	2804
69	precipitation12	41,332	576.98	445.65	45	2048
70	precipitation	41,332	16,680.64	2846.76	11,892	25,596
71	sunshine1	41,332	973.60	387.02	440	1957
72	sunshine2	41,332	954.48	395.54	139	1748
73	sunshine3	41,332	1179.75	250.30	601	1810
74	sunshine4	41,332	1405.16	333.94	639	2097
75	sunshine5	41,332	1356.47	314.98	707	2102
76	sunshine6	41,332	1057.39	302.46	334	1832
77	sunshine7	41,332	1866.88	609.40	626	2918
78	sunshine8	41,332	2061.95	511.96	827	3140
79	sunshine9	41,332	1499.85	387.90	690	2451
80	sunshine10	41,332	1328.47	385.16	318	2392
81	sunshine11	41,332	993.67	311.68	300	1698
82	sunshine12	41,332	1138.37	430.89	324	1917
83	sunshine	41,332	15,811.29	1932.72	10986	19,961
84	water resource	41,332	110.21	58.08	6.93	257.23
85	water supply	41,332	17.36	7.77	1.45	57.38
86	hospitals	42,749	160.27	100.40	28	403
87	hospital beds	42,749	30,391.50	20,020.09	3851	87,950
88	doctors	42,749	20,681.80	13,479.55	2609	57,455
89	insurance	42,749	324.46	193.54	5.21	954.43
90	climate policy	56,970	0.86	0.70	0	10.78

**Table A4:** Performance metrics of Random Forest and XGBoost classifiers on training and validation sets.

Class/Metric	Random Forest (Train)	XGBoost (Train)	Random Forest (Valid)	XGBoost (Valid)
Class 0 Precision	0.8	0.94	0	0
Class 0 Recall	0.05	0.42	0	0
Class 0 F1-score	0.1	0.58	0	0
Class 0 Support	77	77	11	11
Class 1 Precision	0.84	0.85	0.72	0.73
Class 1 Recall	0.96	0.97	0.86	0.86
Class 1 F1-score	0.89	0.9	0.78	0.79
Class 1 Support	15,264	15,264	3864	3864
Class 2 Precision	0.9	0.9	0.43	0.41
Class 2 Recall	0.49	0.55	0.17	0.18
Class 2 F1-score	0.64	0.69	0.25	0.25
Class 2 Support	2484	2484	620	620
Class 3 Precision	0.89	0.91	0.69	0.7
Class 3 Recall	0.83	0.84	0.64	0.64
Class 3 F1-score	0.86	0.87	0.66	0.67
Class 3 Support	9142	9142	2245	2245
Class 4 Precision	0.92	0.95	0.45	0.5
Class 4 Recall	0.36	0.58	0.14	0.29
Class 4 F1-score	0.52	0.72	0.21	0.29
Class 4 Support	849	849	215	215
Accuracy	0.86	0.87	0.7	0.71
Macro Avg Precision	0.87	0.91	0.46	0.47
Macro Avg Recall	0.54	0.67	0.36	0.38
Macro Avg F1-score	0.6	0.75	0.38	0.4
Macro Avg Support	27816	27816	6955	6955
Weighted Avg Precision	0.86	0.88	0.68	0.68
Weighted Avg Recall	0.86	0.87	0.7	0.71
Weighted Avg F1-score	0.85	0.87	0.68	0.68
Weighted Avg Support	27816	27816	6955	6955

**Table A5:** SARIMA Results.

	Coef	Std Err	Z	P > |z	[0.025	0.975]
ar.L1	0.2408	0.351	0.687	0.492	−0.446	0.928
ma.L1	0.1107	0.383	0.289	0.773	−0.640	0.861
ar.S.L12	−0.1659	0.141	−1.178	0.239	−0.442	0.110
ma.S.L12	−0.5302	0.141	−3.769	0.000	−0.806	−0.254
sigma2	0.3394	0.039	8.746	0.000	0.263	0.415
Ljung–Box(L1)(Q):		0.07		Jar que-Bera(JB):		18.23
Prob(Q):		0.80		Prob(JB):	0.00	
Heteroskedasticity(H):		1.19		Skew:	−0.51	
Prob(H)(two-sided):		0.63		Kurtosis:	4.90	
